# Voluntary Ambulation by Upper Limb-Triggered HAL® in Patients with Complete Quadri/Paraplegia Due to Chronic Spinal Cord Injury

**DOI:** 10.3389/fnins.2017.00649

**Published:** 2017-11-21

**Authors:** Yukiyo Shimizu, Hideki Kadone, Shigeki Kubota, Kenji Suzuki, Tetsuya Abe, Tomoyuki Ueno, Yuichiro Soma, Yoshiyuki Sankai, Yasushi Hada, Masashi Yamazaki

**Affiliations:** ^1^Department of Rehabilitation Medicine, University of Tsukuba Hospital, Tsukuba, Japan; ^2^Center for Innovative Medicine and Engineering, University of Tsukuba Hospital, Tsukuba, Japan; ^3^Division of Regenerative Medicine for Musculoskeletal System, Faculty of Medicine, University of Tsukuba, Tsukuba, Japan; ^4^Center for Cybernics Research, University of Tsukuba, Tsukuba, Japan; ^5^Department of Orthopaedic Surgery, Faculty of Medicine, University of Tsukuba, Tsukuba, Japan

**Keywords:** chronic spinal cord injury, complete quadriplegia or paraplegia, gait analysis, rehabilitation, upper and lower limb coordination, hybrid Assistive Limb®

## Abstract

Patients with complete paraplegia after spinal cord injury (SCI) are unable to stand or walk on their own. Standing exercise decreases the risk of decubitus ulcers, osteoporosis, and joint deformities in patients with SCI. Conventional gait training for complete paraplegia requires excessive upper limb usage for weight bearing and is difficult in cases of complete quadriplegia. The purpose of this study was to describe voluntary ambulation triggered by upper limb activity using the Hybrid Assistive Limb® (HAL) in patients with complete quadri/paraplegia after chronic SCI. Four patients (3 men, 1 woman) were enrolled in this study. The mean patient age ± standard deviation was 37.2 ± 17.8 (range, 20–67) years. Clinical evaluation before intervention revealed the following findings: case 1, neurological level C6, American Spinal Cord Injury Association impairment scale (AIS) grade B; case 2, T6, AIS A; case 3, T10 AIS A; and case 4, T11, AIS A. The HAL intervention consisted of 10 sessions. Each HAL session lasted 60–90 min. The HAL electrodes for hip and knee flexion-extension were placed on the anterior and posterior sides of the upper limbs contralaterally corresponding to each of the lower limbs. Surface electromyography (EMG) was used to evaluate muscle activity of the tensor fascia lata and quadriceps femoris (Quad) in synchronization with a Vicon motion capture system. The modified Ashworth scale (mAs) score was also evaluated before and after each session. All participants completed all 10 sessions. Cases 1, 2, and 3 demonstrated significant decreases in mAs score after the sessions compared to pre-session measurements. In all cases, EMG before the intervention showed no apparent activation in either Quad. However, gait phase dependent activity of the lower limb muscles was seen during voluntarily triggered ambulation driven by upper limb muscle activities. In cases 3 and 4, active contraction in both Quads was observed after intervention. These findings suggest that upper-limb-triggered HAL ambulation is a safe and feasible option for rehabilitation in patients with complete quadri/paraplegia caused by chronic SCI.

## Introduction

Patients with complete paraplegia after spinal cord injury (SCI) are unable to stand or walk on their own. Standing exercise for patients with SCI decreases decubitus ulcers, osteoporosis, hip joint flexion, and adduction deformities and improves the performance of the cardiovascular and digestive systems (Karimi, 2011). Conventional gait training using orthoses for complete paraplegia requires locking of the knee joint in an extended position as well as excessive upper limb usage for weight bearing (Karimi, 2011), and it is difficult in cases of complete quadriplegia.

Robotic devices have recently been used in clinical settings for patients with chronic SCI. Exoskeleton robotic devices employed with a treadmill, such as the Lokomat (Hocoma, Switzerland) (Colombo et al., 2000) and LOPES (Veneman et al., 2007) and powered exoskeleton devices, such as the ReWalk (Robotics, Israel) (Miller et al., 2016), have angular sensors in the joints and pelvis as well as foot force pressure sensors. However, they have no sensors to detect the neuromuscular activation of the user.

In sensing a user's neuromuscular activation, our research has focused on another exoskeleton robot, the Hybrid Assistive Limb® (HAL, Cyberdyne Inc., Ibaraki, Japan). HAL is a wearable robot suit that assists the user in voluntary control of knee and hip joint motion by detecting signals from force/pressure sensors in the shoes or even very weak bioelectric signals on the skin surface. Power units on the bilateral hip and knee joints are comprised of angular sensors and actuators, and the control system consists of a cybernic voluntary control (CVC) mode, cybernic autonomous control (CAC) subsystem (Kawamoto and Sankai, 2005), and cybernic impedance control (CIC) mode. The HAL suit has a unique operating system, with a hybrid control system that includes the CVC, CAC, and CIC modes. The CAC mode can automatically move a user's leg using signals from the force-pressure sensors (Ikumi et al., 2017). The CVC mode can support the user's voluntary motion by providing assistive torque to each joint according to their voluntary muscle activity. The CIC mode can adjust to the user's motion while compensating for HAL's weight and joint viscosity.

Gait training with the HAL has been reported to improve gait ability in patients with chronic stroke (Kawamoto et al., 2013; Nilsson et al., 2014; Wall et al., 2015), chronic SCI (Aach et al., 2014; Sczesny-Kaiser et al., 2015; Wall et al., 2015; Ikumi et al., 2017; Shimizu et al., 2017b), and postoperative thoracic ossification of the posterior longitudinal ligament (Sakakima et al., 2013; Kubota et al., 2016, 2017; Fujii et al., 2017) in terms of gait speed, step length, and clinical functional evaluation.

We previously evaluated the application of HAL for single joints in a patient with complete C4 quadriplegia in order to restore elbow joint flexion using residual trapezius muscle activation. We found that the use of HAL that associated the residual muscle contraction to the elbow joint movement might activate the paralyzed muscle (Shimizu et al., 2017a).

We focused on remaining muscle activity in the upper limb in complete paraplegia. Previous studies have reported neural coupling of the upper and lower limbs in humans (Zehr and Duysens, 2004; Dietz, 2011; La Scaleia et al., 2014; Sylos-Labini et al., 2014). Arm-leg coordination was reported to be useful for gait assistive technology (Hassan et al., 2014; La Scaleia et al., 2014). Therefore, we concentrated on the structural analogy and symmetric motion of the shoulder and hip during gait. The lower extremities move synchronously and almost simultaneously with the contralateral upper extremities during natural locomotion. As we flex a shoulder, the contralateral hip flexes; and at the same time the other shoulder extends, with contralateral hip extension.

We hypothesized that triggering lower limb motion by upper limb muscle activity using HAL was feasible for the voluntary generation of gait composed of voluntarily driven hip and knee joint motions, in people with chronic SCI leading to complete paraplegia or quadriplegia with residual control over upper limb movement. In addition, we hypothesized that simulating the synergy of the upper and contralateral lower limbs in voluntary gait during HAL intervention may activate paralyzed lower limb muscles.

Here, we describe the feasibility and effects of upper limb-triggered HAL intervention for patients with complete paraplegia or quadriplegia caused by chronic SCI.

## Materials and methods

### Participants

Four patients (3 men, 1 woman) were enrolled in this study. The mean patient age ± standard deviation was 37.2 ± 17.8 (range, 20–67) years. Clinical evaluation before the intervention revealed the following findings: case 1, neurological level C6, American Spinal Cord Injury Association (ASIA) impairment scale (AIS) (Kirshblum et al., 2011) grade B; case 2, T6, AIS A; case 3, T10, AIS A; and case 4, T11, AIS A. International Standards for Neurological and Functional Classification of Spinal Cord Injury (ISNCSCI) lower extremity motor score (LEMS) was 0 in cases 1, 2, and 3, and 2 in case 4. Participants' clinical data are shown in Table 1.

**Table 1:** Patient characteristics.

**Case**	**1**	**2**	**3**	**4**
Sex	Male	Male	Female	Male
Age	20	67	32	30
Reason for SCI	C3/4 fracture-dislocation	T5/6 Pyogenic Spondylitis	Spinal cord infarction	T12 burst fracture
Post injured period	3y 2 m	2y 3 m	6y 3 m	1y 9 m
AIS	C6 B	T6 A	T10 A	T11 A
Type of paralysis	Spastic	Spastic	Spastic	Flaccid
ISNCSCI UEMS	24	50	50	50
ISNCSCI LEMS	0	0	0	3
History of other gait training	None	None	Long leg brace	Long leg brace
Frequency	1 to 2/month	2/week	1 to 2/month	2/week

This study was conducted in accordance with the Declaration of Helsinki, with approval from the Ethics Committee of the Tsukuba University Faculty of Medicine (approval no.: H26-22). All participants provided written informed consent for participation and publication, including the use of accompanying images.

### HAL intervention

Participants underwent 10 HAL sessions. The frequency of sessions ranged from 2 per week to 1–2 per month. Intervention for cases 1, 3, and 4 was performed on an outpatient basis, and no additional therapies were implemented during HAL intervention. In case 2, HAL intervention was performed as part of an inpatient stay at our hospital in addition to standard physical therapy. Before the intervention, active hip flexion and active knee extension were evaluated using a Trigno™ Lab Wireless electromyography (EMG) system (Delsys Inc., Boston, MA, USA).

### Upper limb-triggered HAL (UT-HAL)

**Figure 1:**
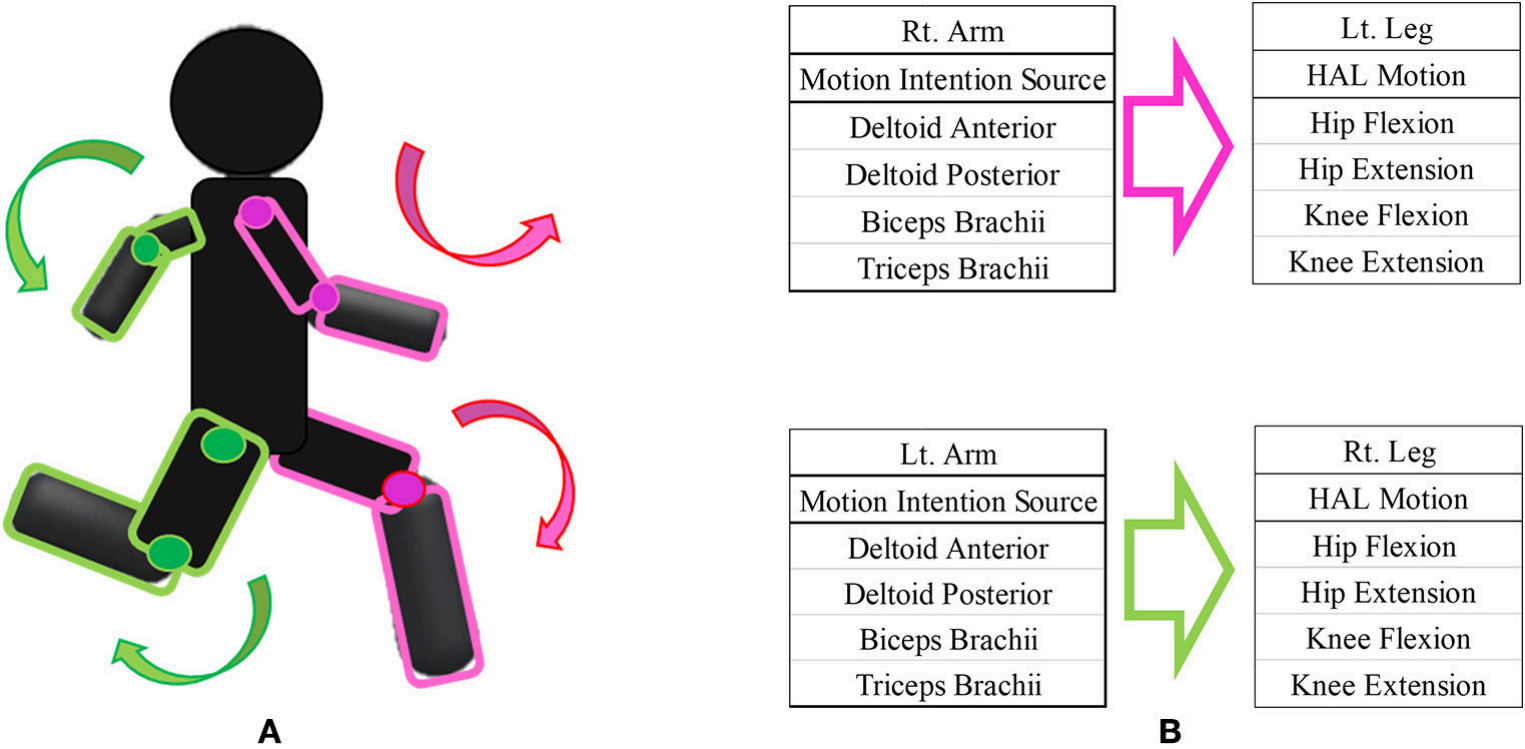
Upper limb-triggered HAL (UT-HAL) method. **(A)** Schema of UT-HAL. **(B)** Picture of patient 1. Muscle activities from the anterior and posterior deltoid for contralateral hip flexion and extension and activities from the biceps and triceps brachii were used for contralateral knee flexion and extension. **(B-1)** Left leg flexion triggered by right arm flexion. **(B-2)** Right leg flexion triggered by left arm flexion.

We designed the HAL intervention based on patients' residual muscle activity. The primary goal of HAL intervention was to achieve voluntary gait using voluntary activation of upper limb motion with the HAL system. On the basis of several previous studies on upper and lower limb coordination in human locomotion (Zehr and Duysens, 2004; Dietz, 2011; La Scaleia et al., 2014; Sylos-Labini et al., 2014), voluntary ambulation with HAL using upper limb activation involved motion intention from the anterior deltoid and posterior deltoid for contralateral hip flexion and extension, respectively, and motion intention from the biceps and triceps brachii for contralateral knee flexion and extension, respectively. We referred to the upper limb-triggered HAL session as the “UT-HAL” method (Figure 1).

The electric motors for the hip and knee joints are controlled in real-time to generate joint torque computed as a weighted difference of the activation of the flexor and extensor muscles. In equation, Thip = Gda^*^Ada-Gdp^*^Adp and Tknee = Gb^*^Ab-Gt^*^At are, respectively, the hip and knee joint torques generated by the motors, where Gda, Gdp, Gb, and Gt are the gain, and Ada, Adp, Ab, and At are the filtered activation of the anterior deltoid, posterior deltoid, biceps, and triceps muscles, respectively. Gda, Gdp, Gb, and Gt are adjusted for the user's comfort.

### Knee extension HAL

The secondary goal of HAL intervention was to regain active knee extension. In patients who were able to flex their hips, a second component was added to each HAL session. In addition to the first component, which involved voluntary gait using the patient's upper limb activation, the second component involved active knee extension using hip flexor activation, focusing on the hip flexor as the site of remaining muscle activation in patients who could not contract the knee extensors.

### HAL session

A typical HAL session lasted 60–90 min, including the time required to attach and detach the device (20 min). The gait session was ~40 min, including a 10-min period of rest. The knee extension session lasted about 30 min including evaluation (10 min) of muscle activity during five repetitions of each of the following motions: active hip flexion; active knee extension; and active combined hip flexion and knee extension, such as in a kicking motion, before and after each HAL session.

A physiatrist was present in case of an emergency, a therapist and two assistants attached and detached the HAL, and an engineer implemented the gait analysis. For safety reasons, a walking device (All-in-One Walking Trainer, Ropox A/S, Naestved, Denmark) with a harness was used to prevent falls.

For participants without a history of ambulation training (cases 1 and 2), we initially used the CAC and CIC mode along with heavy assistance of three therapists, and as they became accustomed to ambulation training, upper limb-triggered methods were introduced.

### Assessments

Assessments were performed before the intervention and during each HAL session. A surface EMG system was used to evaluate the muscle activity of the tensor fasciae lata (TFL) for hip flexion and the femoral quadriceps (Quad) for knee extension on both sides. We chose the TFL for the evaluation of hip flexion because the main hip flexor, the iliopsoas muscle, is located in the deep layer and thus is difficult to detect by surface EMG. We chose the vastus lateralis in the Quad because it is a simple knee extensor. The activity of each muscle was evaluated using EMG collected at 2,000 Hz and filtered with a 30- to 400-Hz band-pass filter using scripts on MATLAB 8.2 (Mathworks, Natick, MA, USA). Motion capture (Vicon MX with 16 T20S cameras, Vicon, Oxford, UK) was used to evaluate foot motion in synchronization with EMG. Following the Vicon plug-in gait marker set, auto-reflective markers were placed bilaterally on the feet, head of the second metatarsal bone of the toe, lateral malleolus of the ankle, and posterior peak of the calcaneus of the heel. The swing phase and stance phase within a gait cycle were extracted according to the movement trajectory of the markers. Heel strikes were detected as the lower peaks of the height of the heel markers, and toe lifts were detected as the lower peaks of the toe markers. The swing phase started with a toe lift and ended with the subsequent heel strike on the same side. The stance phase started with a heel strike and ended with the subsequent toe lift (Ivanenko et al., 2007).

We also evaluated the walking distance and bilateral modified Ashworth scale (mAs) score (range: 0–24; hip abduction, knee extension, and ankle dorsiflexion; Bohannon and Smith, 1987) before and after each HAL session. The mAs scores before and after each HAL session were compared with the Wilcoxon signed-rank test. All analyses were performed using JMP® 10.0.2 (SAS Institute Inc., Cary, NC, USA); *P* < 0.05 were considered significant. Any adverse events associated with HAL intervention were also recorded.

## Results

**Figure 2:**
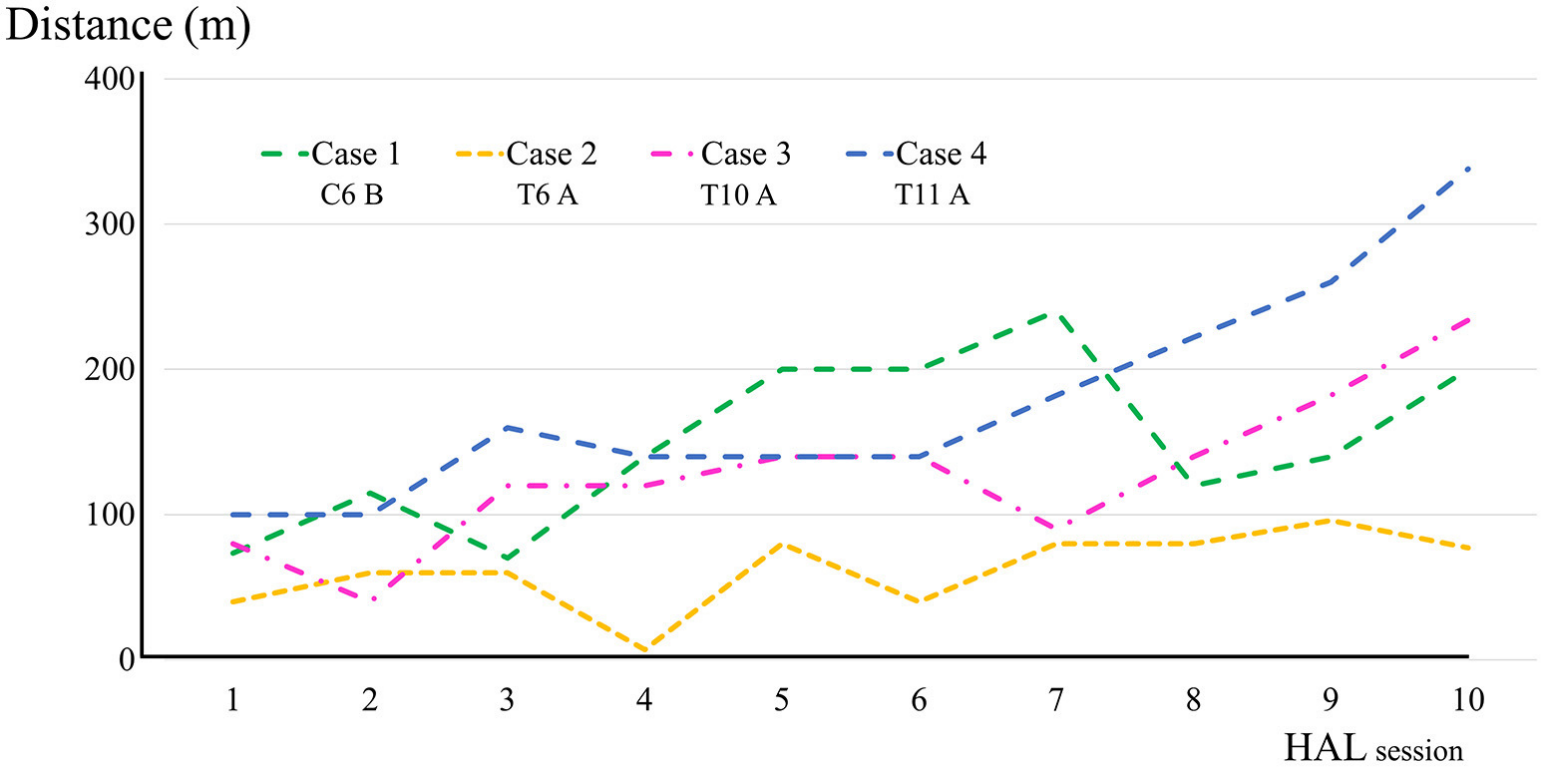
Walking distance in each HAL session in cases 1–4.

**Figure 3:**
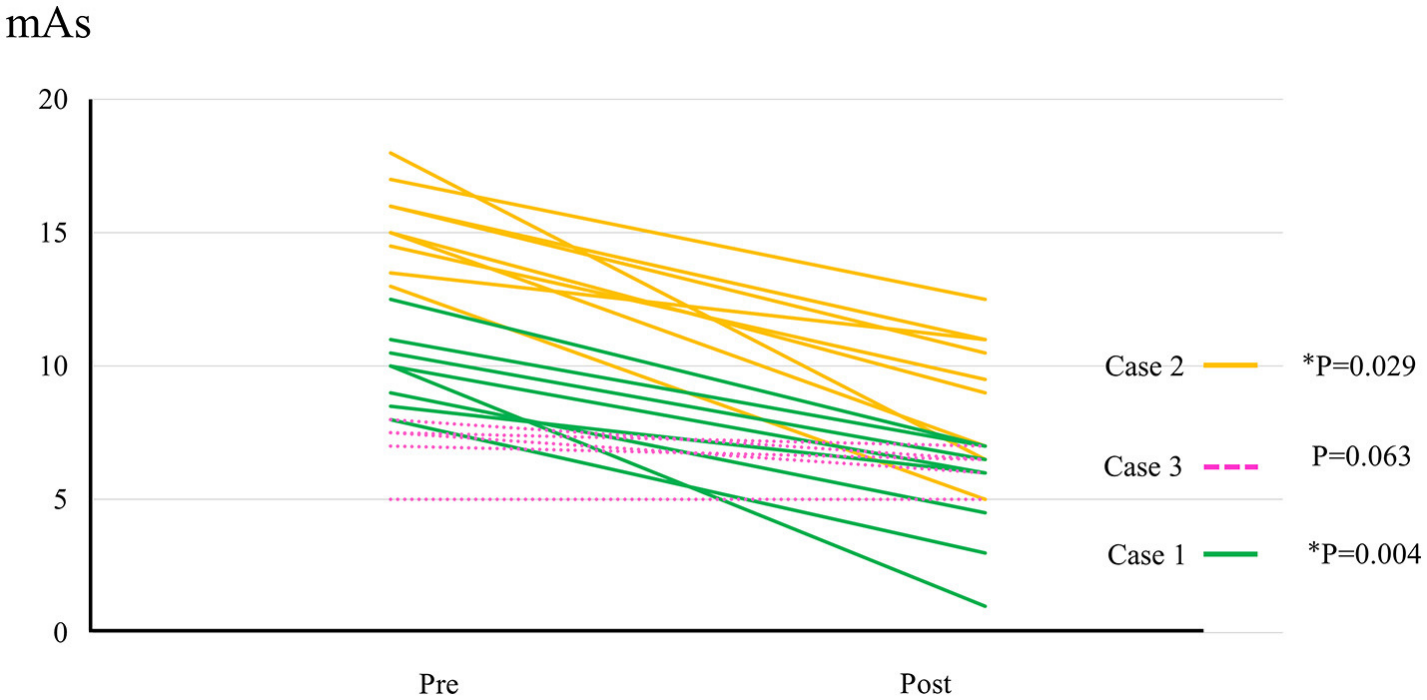
The change of mAs score in cases 1–3. Data evaluated by the same examiner were available from 8 sessions in case 1, 9 sessions in case 2, and 5 sessions in case 3.

**Figure 4:**
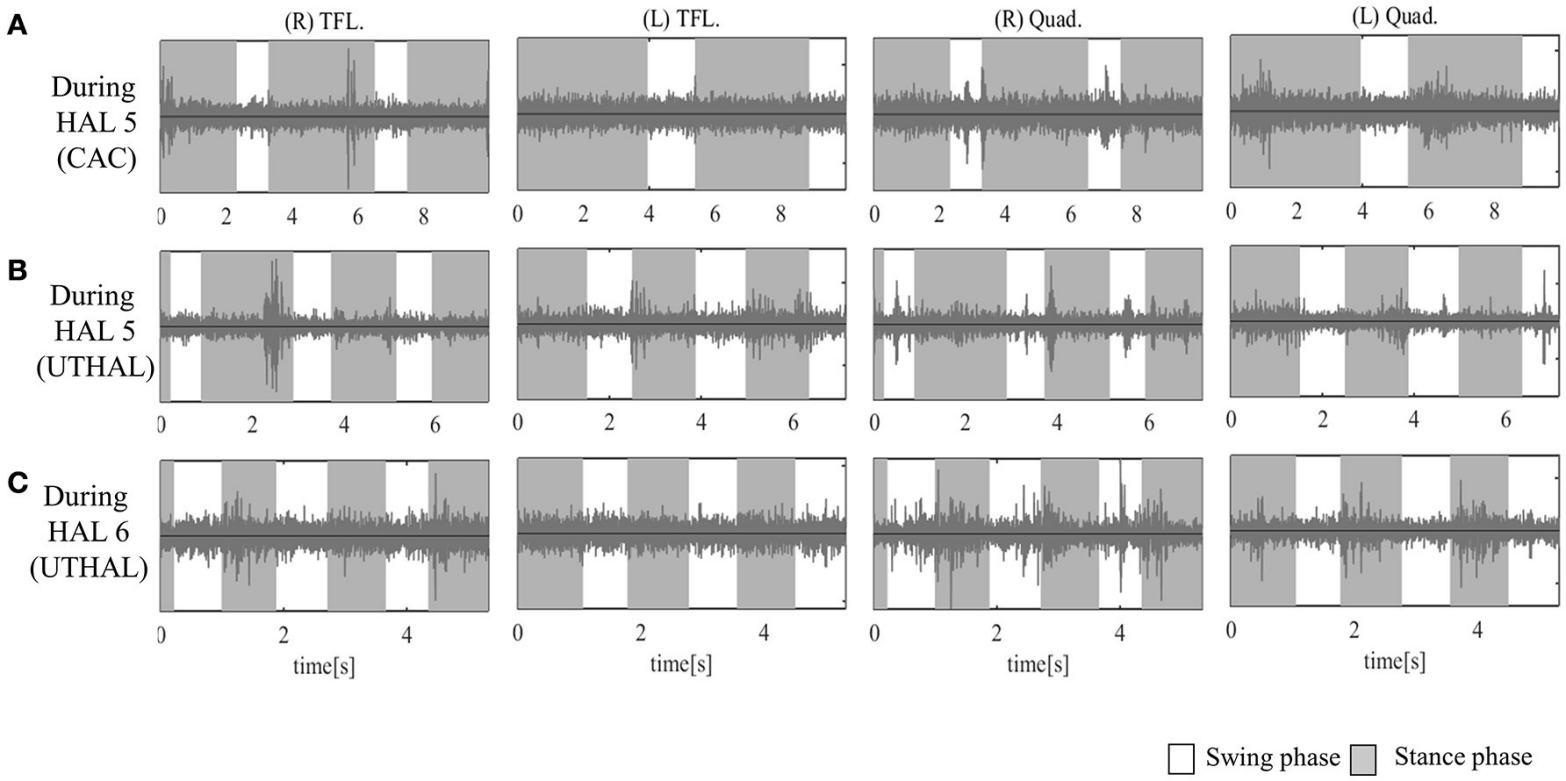
Surface electromyography (EMG) of both TFLs and Quads in case 1. **(A)** During the fifth session using CAC mode, there was some activation in both Quads, and periodic activation only in the left. **(B)** During the fifth session in UT-HAL gait, there was more periodic activation, from the end of the swing phase to early stance phase, than in CAC mode. **(C)** During the sixth session in UT-HAL gait, there was more apparent activation, predominantly on the right side.

**Figure 5:**
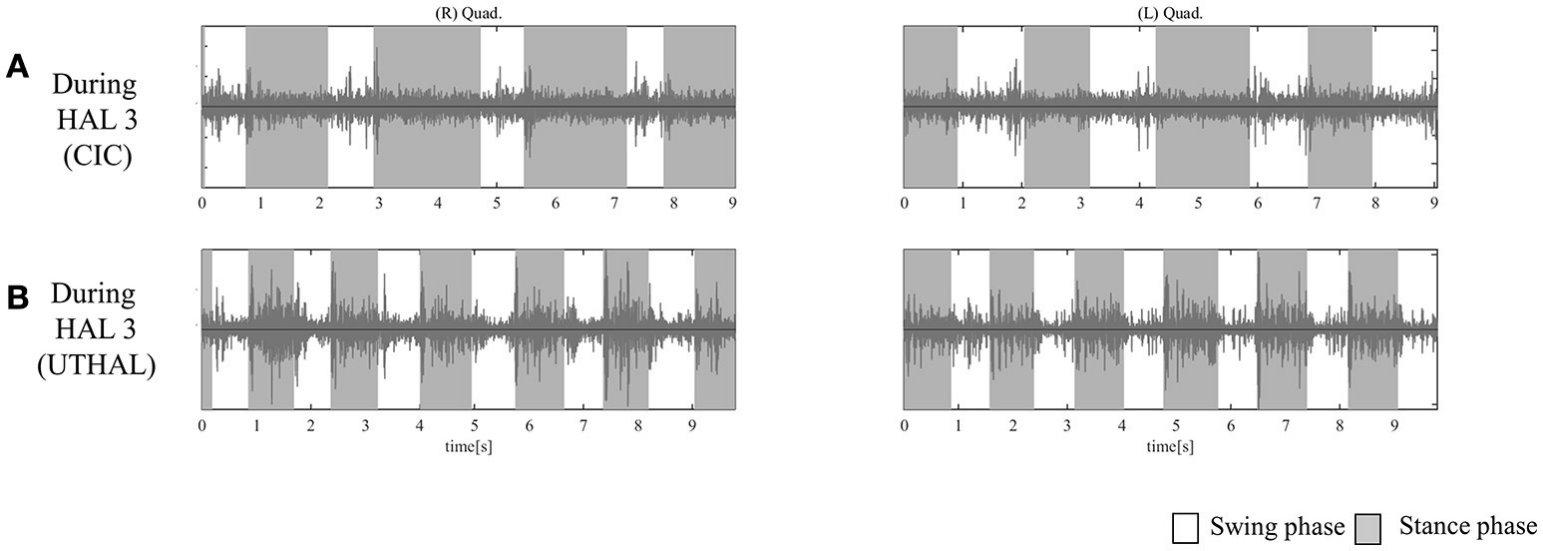
EMG of both Quads in case 2. **(A)** During the third session using CIC mode, there was activation in both Quads in the stance phase. **(B)** During the same third session in UT-HAL gait, there was more apparent activation than during CIC mode.

**Figure 6:**
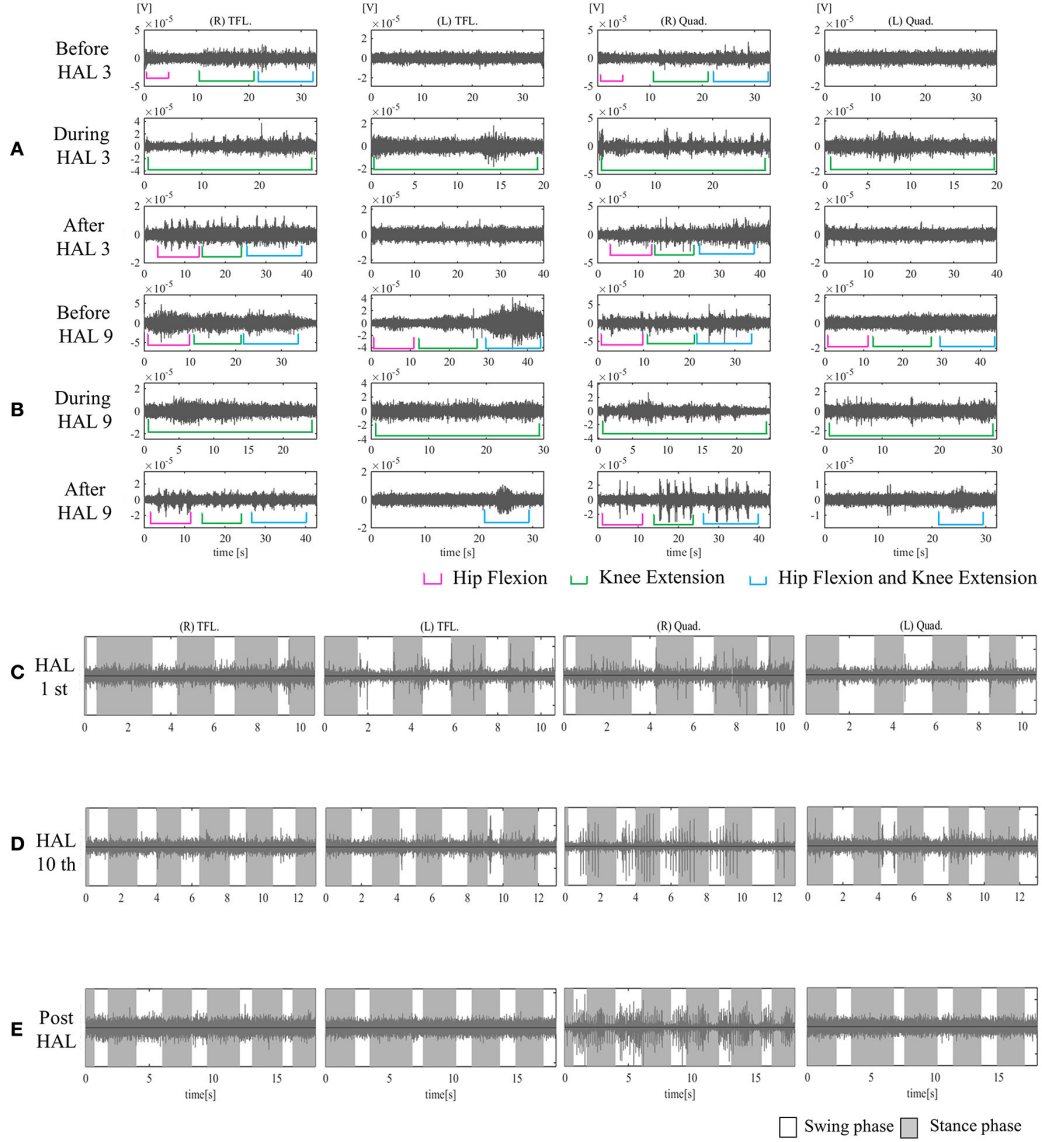
EMG of both TFLs and Quads in case 3. **(A)** Before, during, and after the third knee HAL session. Before this session, there was periodic activation in the TFL and Quad only on the right side. During the HAL session, activation was observed in both TFLs and Quads. After the session, there was rhythmic activation in the right TFL and Quad. **(B)** Before, during, and after the ninth knee HAL session. Before the session, there was periodic activation in both TFLs and the right Quad; during the HAL session, activation was more apparent in both TFLs and Quads; and after the session, more obvious activation was observed in the right Quad, and some activation was seen in the left during hip flexion and extension. **(C)** During the first session in UT-HAL gait, there was some aperiodic activation in both TFLs and the right Quad. **(D)** During the tenth session in UT-HAL gait, there was more rhythmic activation in both Quads during the stance phase, predominantly on the right side. **(E)** During voluntary ambulation with a walker and overhead harness without HAL after the entire intervention, there was rhythmic activation in the right quad from the swing mid-phase to the early stance phase.

**Figure 7:**
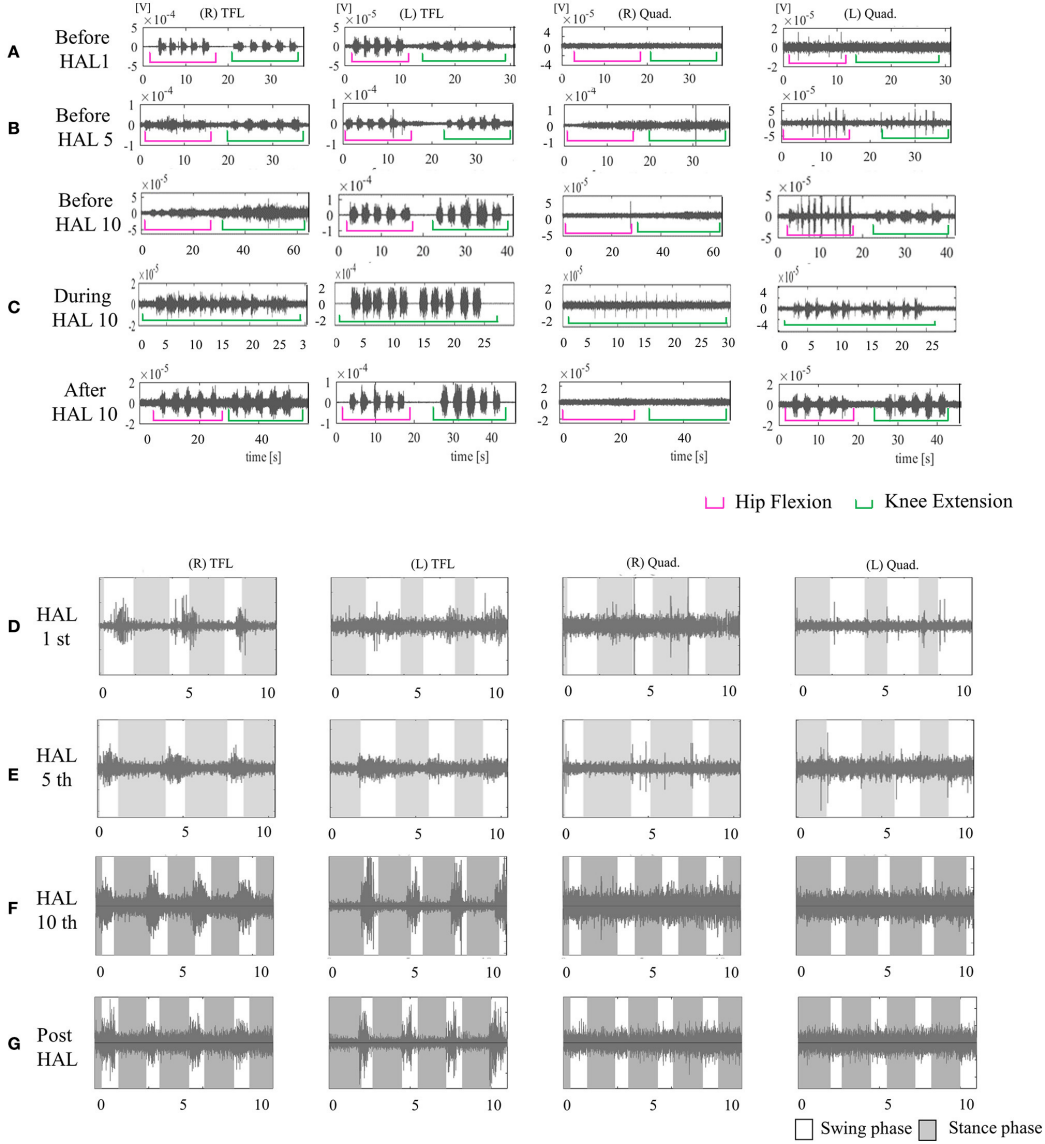
EMG of both TFLs and Quads in case 4. **(A)** Before the first session, there was no voluntary contraction in either Quad. **(B)** Before the fifth session, there was voluntary contraction in the left Quad. **(C)** Before, during, and after the tenth knee HAL session. Before and during the session, there was periodic activation in the left Quad; during the session, the right Quad was also periodically activated. After the session, the left Quad was more periodically activated than before it. **(D)** During the first session in UT-HAL gait. **(E)** During the fifth session in UT-HAL gait. There was activation in both TFLs from the first to tenth sessions; there was no activation in either Quad in the first and fifth gait sessions. **(F)** During the tenth session in UT-HAL gait, there was slight periodic activation during swing phase. **(G)** During voluntary ambulation with a walker and overhead harness without HAL after the entire intervention, there was rhythmic activation in both TFLs, and no periodic activation in either Quad.

All participants completed all 10 sessions. The only observed adverse event was redness caused by contact between the skin and harness in cases 1, 2, and 3 which was avoidable by using a cushion. Figure 2 shows the walking distance in all cases; improvement was observed in cases 3 and 4 as the HAL intervention progressed. The decrease of the mAs score was statistically significant from before to after session; the each post-session mAs score decreased significantly compared to the pre-session mAs score in case 1 (from 9.9 ± 1.5 to 5.1 ± 2.2; *P* = 0.004), case 2 (from 15.3 ± 1.6 to 9.1 ± 2.5; *P* = 0.029), and case 3 (from 7.0 ± 1.2 to 6.2 ± 0.8; *P* = 0.063; Figure 3). In cases 1, 2, and 3, activation was observed in both Quads in the stance phase (Figures 4–**6**), which became more apparent as the sessions progressed. However, in all cases, surface EMG before the intervention showed no apparent activation in either Quad (refer to Figures **6**, **7** for cases 3 and 4). In these graphs, we chose sessions that were representative of the progression of each subject throughout the UT-HAL sessions.

In cases 3 and 4, knee extension sessions were performed using the hip flexor as the trigger of knee extension. In both cases, active contraction in both Quads was observed after intervention. Table 2 shows the neurological findings in all cases after intervention.

**Table 2:** Neurological change after HAL intervention.

**Case**	**1**	**2**	**3**	**4**
AIS before HAL	C6 B	T6 A	T10 A	T11 A
AIS after HAL	C6 B	T6 A	T10 A	T11 C
LEMS before HAL	0	0	0	3
LEMS after HAL	0	0	4	7

## Case descriptions

### Case 1 (Figure 1B)

A 20-year-old male with complete quadriplegia and chronic cervical SCI due to cervical vertebral dislocation (C5/6) underwent HAL intervention with a total of 10 sessions, 1–2 per month, beginning 3 years and 2 months post-injury. He had the ability to perform daily activities independently with the help of a wheelchair, including transfer from/to the wheelchair and intermittent self-catheterization. Before intervention, manual muscle test (MMT) of the upper limb muscles was 5/5 for both deltoids, 5/5 for both biceps brachii, 2/2 for both triceps brachii, and 1/1 for both wrist flexor muscles. The muscle strength of his elbow extensors and wrist flexors had shown recovery; however, his lower limbs had remained in complete paraplegia. AIS was C6 B, ISNCSCI upper extremity motor score (UEMS) was 26/50, and LEMS was 0/50.

On evaluation before HAL intervention, there was no muscle activation in either TFL or Quad. As the patient had no history of ambulation training, in order to become accustomed to ambulation training, he started HAL sessions using CAC or CIC mode accompanied by heavy assistance from three therapists with an overhead harness. Activation in both Quads was observed in the stance phase from the first session. From the latter half of the fifth session, his upper limb was used as a trigger for lower limb movement, and activation became more apparent in the UT-HAL session than in previous sessions. Figure 4A shows surface EMG during the fifth session using CAC mode. There was some activation in both Quads, but no periodic activation in the right Quad. More periodic activation, from the end of swing phase to early stance phase, was observed during UT-HAL gait in the same fifth session (Figure 4B). More apparent activation was present in the sixth session, predominantly on the right side (Figure 4C).

Walking distance increased from 73.4 m (first session) to 200 m (tenth session; Figure 2). The mAs score decreased after each HAL session compared to pre-session measurements; the mean scores before and after the sessions were 9.9 ± 1.5 and 5.1 ± 2.2, respectively (*P* = 0.004; Figure 3).

### Case 2

A 67-year-old male with complete paraplegia and chronic SCI underwent HAL intervention 2 years and 3 months after the onset of complete paralysis caused by pyogenic spondylitis in the fifth and sixth thoracic vertebrae. He was admitted to our hospital to undergo HAL intervention. His main complaint was spasticity. The mAs scores on admission were as follows: total, 16; hip abduction, 3/2; knee extension, 3/2; and ankle dorsiflexion, 3/3; for the right and left sides, respectively. He underwent HAL sessions twice per week. He had the ability to perform daily activities with the help of a wheelchair and his wife, including transfer from/to the wheelchair. Before intervention, AIS was T6 A, ISNCSCI UEMS was 50/50, and LEMS was 0/50. There was no activation in either TFL or Quad.

As the patient had no history of ambulation training, he started HAL sessions using CAC or CIC mode and the heavy assistance of three therapists. Activation in both Quads was observed in the stance phase from the first session. UT-HAL sessions were started from the latter half of the third session, and more apparent activation was observed in the UT-HAL session than during CIC mode (Figures 5A,B).

Walking distance increased from 40 m (first session) to 77 m (tenth session; Figure 2). The mAs score after each HAL session decreased compared to pre-session measurements; the mean scores before and after each session were 15.3 ± 1.6 and 9.1 ± 2.5, respectively (*P* = 0.029; Figure 3).

### Case 3

A 32-year-old female with complete paraplegia and chronic SCI due to spinal cord infarction underwent HAL intervention 6 years and 3 months after spinal cord infarction. She had the ability to perform daily activities with the help of a wheelchair. She had a history of ambulation training using long leg braces. She underwent 1–2 HAL sessions per month.

Before intervention, AIS was T10 A, and ISNCSCI LEMS was 0/50. She was able to contract the right hip adductor slightly. She could not contract either TFL or Quad.

On evaluation before HAL intervention, there was no muscle activation in either TFL or Quad. From the first session, we used the upper limb as the trigger for limb motion. Some aperiodic activation in both TFLs and the right Quad was observed in the first session (Figure 6C).

On evaluation before the second session, she could contract the right TFL; therefore, we added a knee extension session using the right hip flexor as the trigger for right knee extension from the second session.

Figure 6A shows surface EMG in both TFLs and Quads before, during, and after the third knee HAL session. Before the HAL session, there was periodic activation in the right TFL and Quad. During the HAL session, activation was observed in both TFLs and Quads. After the session, there was rhythmic activation in the right TFL and Quad. Before the ninth session, periodic activation was observed in both TFLs and the right Quad; during the HAL session, activation was more apparent in both TFLs and Quads; and after the session, more obvious activation was observed in the right Quad. Some activation was also seen in the left Quad during hip flexion and knee extension (Figure 6B). Figures 6C,D shows surface EMG during the first and the tenth UT-HAL sessions, respectively. In the tenth session, more rhythmic activation was observed in both Quads during the stance phase, predominantly on the right side.

Figure 6E shows surface EMG during voluntary ambulation with a walker and overhead harness without HAL after the entire intervention. Rhythmic activation in the right quad was observed from the swing mid-phase to the early stance phase.

Walking distance increased from 80 m (first session) to 234 m (tenth session; Figure 2). After the intervention, the patient's hip flexor and knee extensor MMT score improved from 0/5 to 1/5 on both sides, and LEMS improved from 0 to 4 (Table 2).

The mAs score was reduced after each HAL session compared to pre-session measurements; the mean scores before and after each session were 7.0 ± 1.2 and 6.2 ± 0.8, respectively (*P* = 0.063; Figure 3).

### Case 4

A 30-year-old male with complete paraplegia and chronic SCI due to twelfth vertebral burst fracture underwent the HAL intervention 1 year and 7 months post-injury. He had the ability to perform daily activities with the help of a wheelchair. He had a history of ambulation training with long leg braces or the assistance of two therapists. He underwent HAL sessions twice per week.

Before intervention, AIS was T11 A, and ISNCSCI LEMS was 3/50. MMT of the hip flexor was 1/5 on the right and 2/5 on the left. There was no voluntary contraction in either Quad (Figure 7A).

From the first session, we used both hip flexors as the trigger for knee extension in the knee extension HAL session, and the upper limb as the trigger of limb motion during the HAL ambulation session. Activation was observed in both TFLs during the gait session, predominantly in the swing phase, but not in either Quad (Figure 7D). On evaluation before the fifth session, voluntary contraction was observed in the left Quad (Figure 7B). Therefore, we placed electrodes at the recommended sites on both limbs in the latter five sessions and performed knee extension sessions triggered by both knee extensors and HAL ambulation sessions triggered by activation of both lower limbs. Figure 7C shows surface EMG in the tenth knee extension session. Before and during the session, there was periodic activation in the left Quad; during the session, the right Quad was also periodically activated. After the session, the left Quad was more periodically activated than before it. Figures 7D–F show EMG in the first, fifth, and tenth HAL ambulation sessions, respectively. There was activation in both TFLs from the first to tenth sessions, and no rhythmic activation in either Quad was observed in the first or fifth gait sessions. During the tenth gait session, there was slight periodic activation during the swing phase.

Figure 7G shows voluntary ambulation with a walker and overhead harness without HAL after the entire intervention. Rhythmic activation was observed in both TFLs, with no periodic activation in either Quad.

Walking distance increased from 100 to 338 m (Figure 2). The mAs score remained 0 before and after each session. After the intervention, the patient's hip flexor MMT score improved from 1/5 to 2/5 on the right, and from 2/5 to 3/5 on the left. Knee extensor MMT also improved from 0/5 to 1/5, and LEMS improved from 3 to 7 (Table 2).

## Discussion

In this study, we focused on residual upper limb muscle activity in complete quadri/paraplegia and coordination between the upper limbs and contralateral lower limbs in natural gait. In addition, we used voluntary contraction of the hip flexors as a trigger for knee extension. We observed neurological changes in two cases after the intervention.

For all cases, including patients with cervical SCI and high thoracic SCI who were considered ineligible to perform conventional walking training with orthoses, it was possible to perform movement of the paralyzed joints with voluntary control of the relevant muscle using HAL. Considering that the method was feasible in case 1, whose injury level was the highest of the among the patients presented in the study, this method is feasible for patients who has at least voluntary control the triceps muscle with MMT score greater than or equal to 1.

The operating system of HAL directly maps in real-time the detected neuromuscular activity to the assistive torque on the joints of the lower limbs, and therefore directly reflects the user's motion intention into the generated motion. By contrast, other exoskeleton robots such as the Lokomat (Colombo et al., 2000), LOPES (Veneman et al., 2007), and ReWalk (Miller et al., 2016) move paretic limbs automatically; therefore, the limbs are passively moved. We focused on exploiting the residual voluntary muscle activation of paraplegia patients. Using the HAL motion assist function in the context of upper-lower limb coordination during gait, voluntary ambulation was possible, and the improvement of muscle activities was observed.

The HAL has been reported to be a feasible tool for some types of neuromuscular disorders (Kawamoto et al., 2013; Kubota et al., 2013, 2017; Sakakima et al., 2013; Aach et al., 2014; Nilsson et al., 2014; Sczesny-Kaiser et al., 2015, 2017; Wall et al., 2015; Fujii et al., 2017; Ikumi et al., 2017; Shimizu et al., 2017b) and to improve ambulation in patients with chronic SCI (Aach et al., 2014; Sczesny-Kaiser et al., 2015; Wall et al., 2015; Ikumi et al., 2017; Kubota et al., 2017; Shimizu et al., 2017b). HAL is able to detect very weak neuromuscular activities, if any, using its surface electrodes and to provide motion assistance. In this sense, HAL is an effective tool for gait training in more severe cases of SCI. However, the conventional method using the CVC mode of HAL is not applicable in patients with complete paraplegia who have difficulty in detecting bioelectrical signals. The CAC mode uses a foot pressure sensor as a motion trigger, and pressure sensors are not effective for patients who cannot place their weight on both feet. Therefore, we chose upper limb motion as the voluntary trigger for lower limb movement during ambulation.

In utilizing upper extremity muscle activation, we focused on the structural analogy and symmetric motion between upper and contralateral lower limbs in natural gait. The upper and contralateral lower limbs move in synchrony and almost simultaneously during natural locomotion. Based on this contralateral relationship of upper and lower limb movement during locomotion, we placed electrodes on the biceps and triceps brachii to drive contralateral knee flexion and extension, respectively, and on the anterior deltoid and posterior deltoid to drive contralateral hip flexion and extension, respectively.

A previous study reported that the paralyzed lower limb muscles of patients with SCI can be activated by passive leg movements, suggesting the residual function of the rhythmic pattern generator circuit after SCI (Kawashima et al., 2005). The rhythmic pattern generator is highly relevant to quadrupedal coordination during locomotion in humans (Dietz et al., 1995; Zehr and Duysens, 2004; Dietz, 2011). In fact, recent research has indicated that voluntary upper limb alternate movement generates locomotor-like movements (Sylos-Labini et al., 2014) and enhances muscle activation in the lower limbs (de Kam et al., 2013), and this property can be used in the control of legged exoskeletons (La Scaleia et al., 2014). From this perspective, we hypothesized that synergistic coordination of the upper and lower limbs during voluntary gait using HAL may activate lower limb muscles.

UT-HAL-assisted gait in SCI patients incorporates coordinated voluntary movement of the multiple joints of the upper and lower limbs. Since UT-HAL connects each of the upper limb muscles to the corresponding lower limb joint movements independently, the coordination of lower limb movement is realized solely by the coordinated voluntary activation of the upper limb muscles. Our hypothesis is that gait phase-dependent, voluntary, and coordinated activation of the upper limb muscles, in coherent combination with the voluntarily generated gait pattern of the lower limbs, may re-activate gait phase-dependent muscle activity in the lower limbs. This is difficult to implement by other assistive devices such as passive orthoses, other robots, or current functional electric stimulation systems (Karimi et al., 2014).

Our protocol included voluntary knee extension. Patients with paralysis of knee extensors use long leg braces in the knee-locked position for walking exercises; therefore, it is difficult for them to train with knee extensor movement during gait exercise. Gait with the HAL enabled users to extend their knee periodically in the stance phase according to the gait cycle without knee locking (Shimizu et al., 2017b).

In case 3, the patient was unable to contract the hip flexor before intervention. However upper limb-triggered HAL ambulation appeared to activate her paralyzed and disused hip flexors. Subsequently, we used improved muscle activation of the hip flexors as the trigger for knee extension.

We speculate that muscle activation acquired during voluntary ambulation using motion intention conferred by residual activity of the upper limb muscle and during voluntary knee extension using residual activity of the hip flexor may contribute to the improvement of paralyzed Quad muscle activation. Both sessions—knee extension with the hip flexor and locomotion with upper limb muscle activation—were effective in improving muscle activity in cases 3 and 4.

In addition, in case 3 (lower panels of Figure 6), the patient contracted the right TFL in the knee extension HAL session, while in the UT-HAL locomotion session the TFL activation was absent. In our opinion, it was easier to contract the TFL in the knee extension session than in the UT-HAL locomotion because during sitting, TFL contraction is accompanied by a simple single-joint motion of hip flexion, and it was easy for her to focus her attention on the motion. On the other hand, while walking, complex coordinated motion involving hip and knee flexion and extension is required, incorporating coordinated activation of multiple muscles. She could generate voluntary gait using UT-HAL and activate the right Quad in phase with the gait; however, she could not achieve the level of coordinated multiple muscle control during gait.

A similar discussion may apply to case 4 (lower panels of Figure 7). Quad activation was present during the knee extension HAL session, while in the UT-HAL locomotion session it was absent. The patient could contract both TFLs and left Quad independently. During walking, he could generate voluntary gait using UT-HAL and activate both TFLs in phase with the gait. However, he could not reach the level of including the newly activated left Quad in the coordinated activation of the muscles. We consider HAL intervention was effective for achieving voluntary contraction of single-joint muscles; however, a longer-term intervention might have been necessary in order for this patient to be able to perform coordinated muscle control including the newly activated muscles during gait.

Cases 1 and 2 had not experienced conventional walking training before the HAL intervention. They had complete paralysis in both lower extremities; however, during HAL ambulation, there was some activation in the TFLs and Quads. In addition, there was more apparent activation in UT-HAL sessions than in CAC sessions. We consider that ambulation with voluntary control have positively influenced periodic activation.

After intervention, the neurological levels in cases 1 and 2 were unchanged; however, in these cases, spastic paralysis evaluated by the mAs score after each session decreased from pre-session values. As spasticity was the main concern in case 2, this change represented meaningful clinical data. Regarding the reduction of spasticity, we previously reported a decrease of mAs after HAL gait training for a patient with C4 SCI (Ikumi et al., 2017). Spasticity negatively influences quality of life for many SCI patients by causing pain, joint contracture, and restricted activity of daily living (Adams and Hicks, 2005; van Cooten et al., 2015). Therefore, this method may be useful to reduce spasticity and related concerns.

One limitation of this protocol is that it is not suitable for patients who are unable to control upper limb muscles. In addition, it is challenging for patients with severe joint deformities or orthostatic hypotension. It is important for medical staff to evaluate skin problems or bruising in paralyzed areas because of their sensory deficiency. Moreover, although neurological improvement was observed in two cases in this study, we did not confirm the underlying mechanism behind this change. Future perspectives include neurological evaluation of this protocol using, for example, functional MRI of the spinal cord (Stroman et al., 2004) or spinal cord mapping of motor activity during gait (Ivanenko et al., 2008).

The ambulation protocol using HAL and voluntary upper limb muscle activation was feasible for complete paraplegic patients. Moreover, patients with injury to the cervical cord or upper thoracic cord, who have difficulty performing conventional gait training with orthoses, may experience decreased spasticity and activation of paralyzed muscles. It is applicable for patients with both spastic and flaccid-type paralysis and represents a novel option for such patients to perform voluntary controlled ambulation similar to natural gait by using their residual neuromuscular activities. Therefore, this HAL protocol may contribute to broadening gait rehabilitation for complete SCI patients.

## Conclusions

This study reports the safety and feasibility of upper-limb-triggered HAL ambulation for patients with complete quadri/paraplegia caused by chronic SCI. Improvement of activation in both TFLs and Quads was observed for two cases, and decreased spasticity after each session was also observed for three cases with spastic-type paralysis. Therefore, voluntary upper limb muscle activation using HAL is a potential option for rehabilitation in complete quadri/paraplegic patients.

## Author contributions

All authors participated in the design, execution, and analysis of these studies and have seen and approved the final version of the manuscript. YuSh and HK participated in the study design and drafted the manuscript. YuSh, HK, SK, and YuSo executed HAL interventions and performed the data analysis. YoS conceived the device and helped to draft the manuscript. KS, TU, TA, YH, and MY participated in the study design and helped to draft the manuscript. MY is the principal investigator of this study and participated in the design and coordination of the study. Furthermore, this manuscript has been revised by a professional editor whose first language is English.

### Conflict of interest statement

YS is the C.E.O., shareholder, and director of CYBERDINE Inc., which produces the robot suit HAL. CYBERDINE was not involved in study design, data collection, analysis, writing or submission of this article. The other authors declare that the research was conducted in the absence of any commercial or financial relationships that could be construed as a potential conflict of interest.
